# Spatial and seasonal variation in thermal sensitivity within North American bird species

**DOI:** 10.1098/rspb.2023.1398

**Published:** 2023-11-08

**Authors:** Jeremy M. Cohen, Daniel Fink, Benjamin Zuckerberg

**Affiliations:** ^1^ Department of Forest and Wildlife Ecology, University of Wisconsin, Madison, WI, 53706, USA; ^2^ Department of Ecology and Evolutionary Biology, and; ^3^ Center for Biodiversity and Global Change, Yale University, New Haven, CT, 06520, USA; ^4^ Cornell Lab of Ornithology, Cornell University, Ithaca, NY, 14850, USA

**Keywords:** birds, citizen science, climate change, eBird, macroecology, species distribution models

## Abstract

Responses of wildlife to climate change are typically quantified at the species level, but physiological evidence suggests significant intraspecific variation in thermal sensitivity given adaptation to local environments and plasticity required to adjust to seasonal environments. Spatial and temporal variation in thermal responses may carry important implications for climate change vulnerability; for instance, sensitivity to extreme weather may increase in specific regions or seasons. Here, we leverage high-resolution observational data from eBird to understand regional and seasonal variation in thermal sensitivity for 21 bird species. Across their ranges, most birds demonstrated regional and seasonal variation in both thermal peak and range, or the temperature and range of temperatures when observations peaked. Some birds demonstrated constant thermal peaks or ranges across their geographical distributions, while others varied according to local and current environmental conditions. Across species, birds typically demonstrated either geographical or seasonal adaptation to climate. Local adaptation and phenotypic plasticity are likely important but neglected aspects of organismal responses to climate change.

## 1. Introduction

Anthropogenic climate change is impacting wildlife across organizational levels, from individuals to populations to species [1], representing a leading conservation priority for wildlife management [2,3]. Using traditional species distribution models (SDMs) or ecological niche models, ecologists typically operate at the species level to quantify responses to the thermal environment and predict the consequences of climate change [4]. These approaches generally ignore adaptive capacity and phenotypic plasticity within species, implicitly assuming that thermal sensitivity, or the influence of temperature on behaviour, performance or fitness, is static in both space and time [4,5]. However, emerging physiological evidence suggests that populations of a species are locally adapted to distinct thermal conditions depending on the climate zones they inhabit, and individuals may dynamically alter their thermal sensitivity to respond to local or seasonal conditions via phenotypic plasticity [6–8]. Standard approaches assuming constant thermal sensitivity across continental ranges and the full annual cycle may thus be inadequate to describe the full spectrum of responses to climate change exhibited by a species [9]. As organisms increasingly face novel climates, understanding variation in thermal sensitivity within species will provide more detailed insights about when and where organisms are most impacted [4,7].

**Figure 1.**
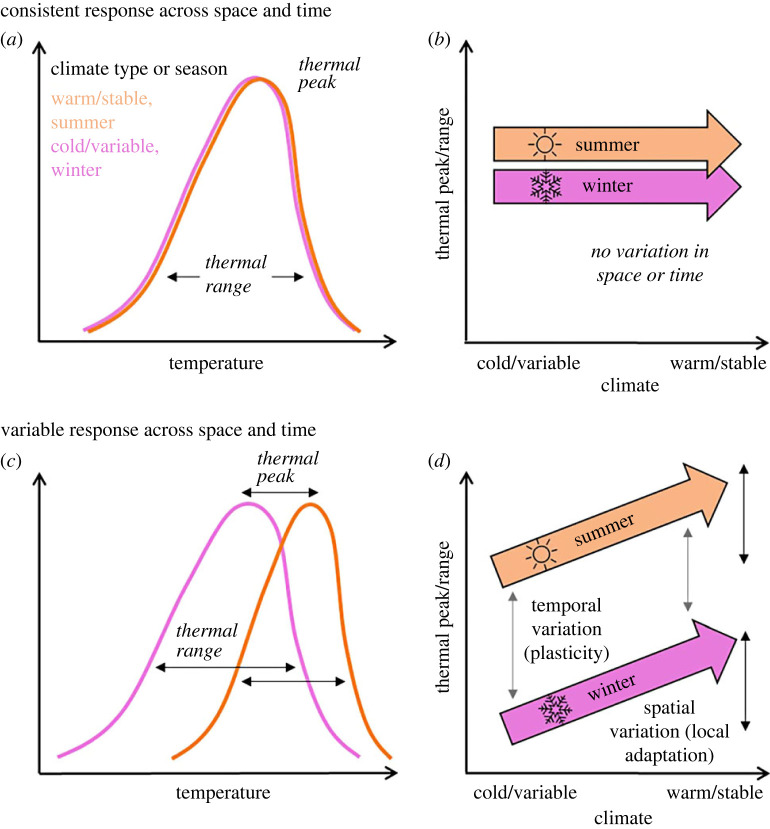
Local and seasonal variation in thermal sensitivity within species. Conceptual schematic of spatial and seasonal patterns in the thermal sensitivity of a species given (*a,b*) the assumption of a lack of variability in thermal sensitivity within a species, and (*c,d*) variation in space and time due to local adaptation and phenotypic plasticity, respectively. In (*a,b*), curves represent hypothetical relationships between temperature and activity levels in either a warm, stable climate or during summer (orange curve) or a cold, variable climate or during winter (purple curve). Thermal peak represents the temperature of peak activity, while thermal range represents the range of temperatures at which activity is high. Northern and winter thermal ranges are expected to be greater than southern and summer ranges owing to the greater variability in available conditions. In (*b,d*), variation in thermal peak and thermal range is driven by climate context when intraspecific variation is present. Orange arrows represent summer trends and purple arrows winter trends. Black arrows, mirroring the slopes of the thermal response across space, represent the degree of spatial variability in thermal peak or range, while grey arrows reflect the distance between summer and winter trends and represent seasonal variability.

Populations within a species are likely to exhibit local and seasonal variation in their responses to thermal conditions owing to physiological mechanisms and constraints ([6–8,10–12]; figure 1). Across geographical gradients, populations of wide-ranging species are likely adapted to local climatic conditions [8,13,14]. Physiological studies have suggested that, within species, southern and lowland populations adapted to warm climates demonstrate warmer optimum thermal performance temperatures when compared with northern and high-elevation populations, which demonstrate cooler optimums [15,16]. Thermal breadth, or the range of tolerable conditions, is associated with variability in the local climate, with ‘thermal specialists' being found in more stable climates and ‘thermal generalists' in more variable climates [17,18]. Given that climatic variability is increasing with climate change [19–21], these findings highlight the importance of considering population-level variation in thermal breadth [8]. In seasonal environments, non-migratory organisms must adapt to seasonally variable weather via phenotypic plasticity, often undergoing behavioural and physiological changes (foraging during different times of day, seeking out refugia, gaining fat reserves etc.) to cope with cold winter temperatures [22,23]. Indeed, physiological studies have revealed that organisms often fluctuate in thermal sensitivity depending on time of year [24].

Together, evidence from physiological and behavioural studies suggests that local and seasonal variability in thermal sensitivity driven by adaptation and plasticity may be common, especially among species occurring across latitudinal or elevational gradients or occupying highly seasonal environments [6,7]. However, it remains unclear whether variability in thermal sensitivity exists across large numbers of species and if its extent is predictable based on species' functional traits or phylogenetic relatedness [7]. Thus, studies are needed to determine whether species demonstrate consistent thermal sensitivity across their ranges, or whether sensitivity is typically tuned to the local environment and season. Field- or laboratory-based studies conducted with one or a few species (i.e. most physiological and behavioural studies) can rarely estimate this variability across species occupying broad geographical regions or the extent to which it is associated with certain traits across many species. To characterize regional and seasonal variation in behavioural responses to temperature, these relationships can be modelled at high temporal resolutions (to capture dynamic changes in temperature) across multiple seasons, and with high spatial resolution across broad geographical extents.

Across species, regional and seasonal variation in thermal sensitivity may be mediated by morphological or life-history traits [25]. For example, local adaptation and phenotypic plasticity may be less important for larger-bodied species, which retain heat more effectively (Bergmann's Rule; [26]), and species with larger appendages which are important for effective heat dissipation (Allen's Rule; [27]). Habitat generalists, which may have more thermal flexibility than generalists [28], and species occupying forested or urban habitats, which have more microclimates to buffer environmental conditions than open/grassland species [29], may be likely to exhibit local adaptation and seasonal plasticity. Thus, we hypothesize that adaptation and plasticity is greatest in birds that (1) are small-bodied, (2) have smaller appendages, (3) are habitat generalists, and (4) occupy forested or urban habitats. Understanding which species have greater adaptation and plasticity in thermal sensitivity—including both thermal peak and range—is an important step towards anticipating organismal responses to climate change. For such species, a cold-adapted northern population may be more sensitive to warming events than a warm-adapted southern population, and a population from a stable climate may be more sensitive to increasing temperature variability than a population from a variable climate.

Here, our goal was to analyse how sensitivity to the thermal environment varies across species' ranges and the annual cycle. Unlike traditional species distribution or niche modelling approaches, which treat species' associations with thermal conditions as static across space and time, our ensemble modelling approach characterized responses that can spatio-temporally vary. Specifically, our approach iteratively models the local associations between species observations throughout the annual cycle (i.e. occurrence rate) and daily temperature. Daily changes in rates of observations represent behavioural activity in response to temperature fluctuations [30] (figure 1). We measured thermal sensitivity as both thermal peak, or the temperature at which occurrence rates are greatest, and thermal range, or the range of temperatures at which a species occurs often (table 1), analogous to the physiological properties of thermal optimum and thermal breadth, respectively. If a species exhibits a similar response to temperature throughout its range, it likely lacks population-level variability in thermal sensitivity, and if the response varies over space, it likely has population-level variability. We interpret spatial variation in thermal peak or range within a species to be evidence of local adaptation (rather than phenotypic plasticity), given the evidence for this phenomenon in controlled physiological experiments. Meanwhile, we interpret seasonal variation in thermal peak or range to be evidence of phenotypic plasticity, given that similar sets of individuals are present at each location across the annual cycle.

**Table 1.** Terms relevant to estimating spatio-temporal variation in thermal sensitivity.

term	statistical definition	biological definition
thermal peak	the temperature at which occurrence is predicted to be greatest within the stixel (local modelling area)	the temperature at which a population is most likely to be observed
thermal range	the difference between the value of daily temperature above the stixel's thermal peak at which predicted occurrence falls below 80% of the maximum value and the value below the thermal peak at which occurrence falls below 80% of the maximum value	the range of temperatures at which a population is observed often
spatial variation	the slope between thermal peak or range and mean annual temperature or mean annual temperature range, respectively, across all stixels spanning a geographical–climatic gradient within the given season (summer or winter)	the extent to which thermal sensitivity either reflects local adaptation (values closer to 1) or is similar across regions (0)
seasonal variation	the mean stixel-level difference in thermal peak or range between seasons	the extent to which thermal sensitivity is variable over time, reflecting phenotypic plasticity at the population level (high values) or consistency across seasons (0)

We used North American bird species as a case study because they are highly detectable and demonstrate strong sensitivity to weather and climate [31]. Moreover, enormous quantities of observational data are available for birds across large regions over both space and time, important for the detection of subtle changes in occurrence over varying temperatures. We focused on 21 bird species that met the following criteria: (1) broad ranges spanning latitudinal and climate zones, enabling comparisons of populations occupying diverse climates; (2) year-round presence in most of their ranges for most populations, enabling direct comparisons of similar populations over seasons; and (3) highly overlapping ranges among multiple species, minimizing variation in available thermal conditions between species that could account for differing thermal responses. Thus, if species demonstrate different levels of local and seasonal variation in thermal response despite highly similar ranges, this is a consequence of an organismal response to temperature and not simply a reflection of available conditions.

Specifically, we pose the following questions:

1. Do birds vary in thermal sensitivity across their ranges, suggesting local adaptation?
2. Do birds vary in thermal sensitivity across summer and winter seasons, suggesting phenotypic plasticity?
3. Do species that vary in thermal sensitivity locally also do so seasonally? Species that exhibit high spatial and seasonal variation may have increased adaptive capacity whereas a negative relationship suggests a trade-off (e.g. a species with high plasticity is less reliant on local adaptation).
4. Is variation in responses to thermal conditions mediated by species' traits or phylogeny?

**Figure 2.**
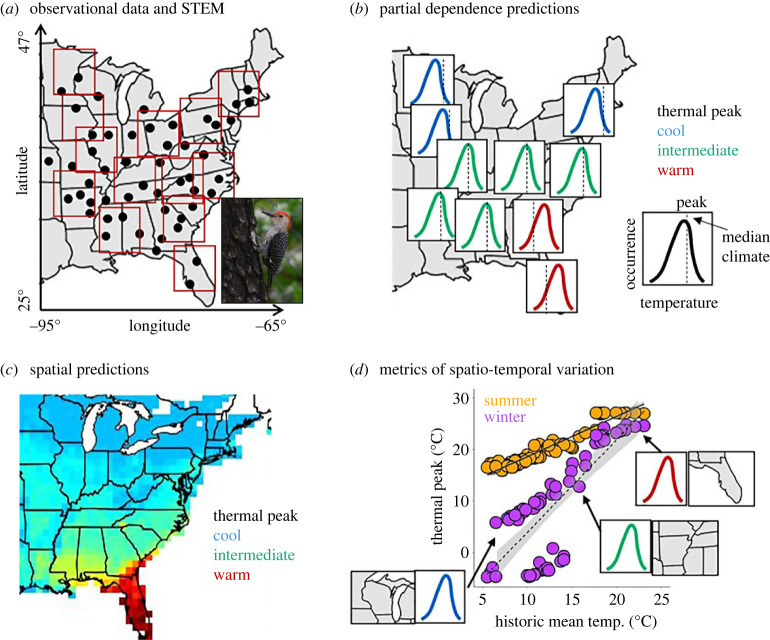
Estimating thermal sensitivity across space and time. Conceptual diagrams outline the process for estimating spatial and temporal variation in thermal sensitivity. (*a*) Relationships between weather and hypothetical observations of an example species (red-bellied woodpecker, *Melanerpes carolinus*; points) are modelled using local species distribution models within smaller geographical regions, known as ‘stixels’ (red boxes), using spatio-temporal exploratory models (STEMs), an ensemble modelling approach. Note that the true size and number of stixels in our analyses is not accurately represented in the conceptual diagram. (*b*) Within each local model, partial dependence statistics predict relationships between temperature and occurrence, and thermal peak is derived from the temperature at which occurrence is predicted to be greatest. Predictions are repeated assuming different times of year. (*c*) Predictions of thermal peak in space are generated for all pixels by averaging approximately 20 predictions from overlapping stixels. (*d*) Thermal peaks (points) based on each stixel and season (orange, summer; purple, winter) are plotted according to their historic mean climate and thermal peak. Trendlines (solid, summer; dashed, winter) and grey shading represent seasonal linear best fit and 95% confidence intervals. Insets demonstrate examples of regional partial dependence predictions corresponding to several points.

To address our questions, we developed an analytical framework for exploring variation in thermal sensitivity based on observational data from eBird, a citizen science initiative in which users submit bird sightings [32]. eBird is especially useful for our approach because it has a massive data volume (over 500 million US records) with dense coverage, and observations are collected throughout the year [33]. We leveraged this dataset to identify regional and seasonal variation in thermal sensitivity for 21 species, fitting SDMs within a spatio-temporal exploratory model (STEM) wrapper [34,35] (figure 2). STEM is an ensemble modelling approach that fits local SDMs over broad spatial extents, allowing spatial variation in relationships between weather conditions and observations. We fitted models using data across the full annual cycle and generated predictions for both the summer and winter seasons. Thus, we quantified associations between observations and daily temperature at local and seasonal scales to assess thermal responses across a continental extent encompassing approximately 900 million km^2^ (figure 2). Finally, we examined species-level trait and phylogenetic associations with intraspecific variation in thermal responses over both space and time.

## 2. Material and methods

### (a) eBird observational data

We compiled all ‘complete checklists' contributed to eBird in much of the contiguous USA and southern Canada (bounding box with dimensions 25° to 47° N and 60° to 125° W) between 2004 and 2018. When submitting ‘complete checklists', users indicate that all identified species were recorded, allowing the inference of non-detection for presence–absence modelling. We applied filters to the data in accordance with established best practices [36]. We limited checklists to ‘travelling' or ‘stationary' observations, excluding exhaustive area-counts, which are less numerous and not directly comparable with most eBird data. In all checklists, subspecies information was discarded, and observations were summarized at the species level. Likewise, we excluded checklists with high values of effort (greater than 3 h or greater than 5 km travelled, to mitigate positional uncertainty in eBird data) or extreme checklist calibration index (CCI) scores (*z*-score less than −4 or greater than 4), an index designed to capture inter-observer variation among checklists [36]. To mitigate site selection and temporal bias, we also filtered checklists by randomly selecting one observation per 5 km^2^ grid cell during each week [36]. Database management was completed using *tidyverse* [37]. Although the number of observations may differ seasonally in some regions, our presence–absence modelling approach primarily considers the proportion of positive to negative occurrence records and so variation in the total number of observations is relatively unimportant.

### (b) Species distribution models: environmental covariates

We included environmental features in each model to account for the many factors that influence species' detection and occurrence rates. To account for variation in detection rates associated with search effort and among observers, we included time spent birding, number of birders, whether a checklist was categorized as travelling or stationary, distance travelled and CCI as features in SDMs (see below) following established best practices [36]. Further, we accounted for seasonal and daily timing by including calendar date and the time difference from solar noon in models.

To account for species preferences in landscape composition and configuration, we gathered land and water cover and topographic data corresponding to each checklist. We obtained annual landcover data from the Moderate Resolution Imaging Spectroradiometer (MODIS) Land Cover Type (MCD12Q1) Dataset, version 6 (https://lpdaac.usgs.gov/dataset_discovery/modis/modis_products_table/mcd12q1). For each checklist, we calculated the proportion of land and water classes within a 1.4 km radius [38], including grasslands, croplands, mixed forests, woody savannahs, urban/built, barren, evergreen broadleaf, evergreen needle, deciduous broadleaf, deciduous needle, closed shrubland, open shrubland, herbaceous wetlands and open savannah. Land-cover data varied annually, although we used 2017 land-cover values for checklists recorded in 2018. We also collected topographical information (median aggregations of elevation, eastness, northness, roughness and topographic position index (TPI) at a 1 km^2^ resolution) from the Global Multi-Terrain Elevation Dataset, a product of the US Geological Survey and the National Geospatial-Intelligence Agency [39].

Daily mean temperatures and total daily precipitation corresponding to each checklist were compiled from Daymet, a high-resolution, interpolated grid-based product from NASA offering daily, 1 km^2^ scale weather data across North America [40]. Daily mean temperatures were estimated from the mean of minimum and maximum daily temperatures. To account for the climate zone of each observation point, we included mean temperature and precipitation (via Worldclim; [41]) as additional features in random forests (RFs). The spatial resolution of our environmental features is similar to the typical radius of search effort in checklists within our filters (T. Auer 2021, personal communication).

### (c) Species distribution models: random forest

The objective of the analysis was to study the relationship between species' local occurrence rates and daily temperature for widespread, commonly detected species. We modelled responses to daily temperature in species with sufficient data to detect regional-scale variation in the relationships between temperature and occurrence rate across the study extent. We excluded long-distance migratory species from our analysis because winter and summer populations at the same locations are not directly comparable, although our species do move semi-locally within our spatial extent. Within the eastern or western USA and Canada, we selected species with mostly sympatric ranges to ensure that species-level differences in spatial and seasonal thermal sensitivity were not due to differences in the availability of weather conditions across species. We divided the continent in this way to increase the similarity and overlap between species' range extents. In the east (less than 100° W), we modelled northern cardinal (*Cardinalis cardinalis*), blue jay (*Cyanocitta cristata*), American crow (*Corvus brachyrhynchos*), mourning dove (*Zenaida macroura*), white-breasted nuthatch (*Sitta carolinensis*), black-capped chickadee (*Poecile atricapillus*), Carolina chickadee (*Poecile carolinensis*), tufted titmouse (*Baeolophus bicolor*), Carolina wren (*Thryothorus ludovicianus*), downy woodpecker (*Dryobates pubescens*), hairy woodpecker (*Dryobates villosus*), red-bellied woodpecker (*Melanerpes carolinus*) and northern mockingbird (*Mimus polyglottos*). In the west (greater than 100° W), we modelled mountain chickadee (*Poecile gambeli*), chestnut-backed chickadee (*Poecile rufescens*), pygmy nuthatch (*Sitta pygmaea*), Bewick's wren (*Thryomanes bewickii*), black-billed magpie (*Pica hudsonia*), Steller's jay (*Cyanocitta stelleri*), Anna's hummingbird (*Calypte anna*) and acorn woodpecker (*Melanerpes formicivorus*).

For each species, we fitted a set of dynamic SDMs using RF (*ranger* package; [42]), a flexible machine learning method used in a number of species distribution modelling problems [43] and designed to analyse large datasets with many features, adjust automatically to complex, nonlinear relationships and consider high-order interactions between features. Dynamic SDMs account for temporally varying predictor variables, such as weather, to influence predictions of species distributions, rather than assuming that these environmental features are constant. To detect spatio-temporal variation in species responses to climate across broad spatial extents, we fitted RF models within spatio-temporal exploratory models (STEMs) as a wrapper [34,35]. We used STEMs to generate a randomized ensemble of partially overlapping regional models consisting of 10° × 10° cells (stixels) across our spatial extent (figure 2) and fitted independent RF models within each cell with a minimum of 20 000 checklists, producing a uniformly distributed ensemble of hundreds of partially overlapping models. Within each stixel, we assume relationships between species' occurrence and environmental variables to be stationary. We generated spatially explicit occurrence estimates by averaging predictions from all regional RFs overlapping a given location. STEM is an effective method for measuring non-stationary relationships between environmental features and observations [29,35,44,45].

Before modelling, all data were split 75/25 into training/testing subsamples. Initial training data were further split 75/25 for model training and validation. For each set, we equalized weighting by year, accounting for the increasing sample sizes by year generated by eBird (submissions increase 30% annually). For each model, we calibrated predicted probabilities based on a validation set calibration adjustment. Finally, we assessed the fit of each model based on a series of predictive performance metrics computed by comparing model predictions against the withheld test data, including specificity, sensitivity, kappa (a measure of model performannce) and area under the curve.

### (d) Detectability and influence of human observers

Our analyses consider variation in detectability among checklists by including variation in observer distance, duration of observation, number of observers, and variation among observers, established best practices to account for detection in observational eBird data analogous to an occupancy model [46]. However, it remains possible that detectability of a given species by a human observer may vary across thermal conditions as well. We explored the possible confounding influence of daily temperature on eBird observers by fitting an RF model with daily temperature as the dependent variable, and effort, CCI, landcover, topography, and mean climate features and all model parameters identical to our primary models. We then examined the explanatory power of this model using root mean squared error (RMSE), Spearman's rank correlation, and the partial dependency of daily temperature based on effort variables and CCI.

### (e) Partial dependence and variability metrics

To examine the regional-scale relationships between species occurrence rates and daily mean temperature, we calculated the partial dependence [47] within each stixel. Partial dependence statistics describe how occurrence varies as a function of certain focal features, such as temperature, averaging across the values of all other features in models (except date, see below). By averaging, partial dependence estimates capture systematic changes in occurrence rates associated with temperature while controlling for all other sources of variation captured by the models, including heterogeneity in search effort and among observers. For each species, we generated partial dependence estimates for both summer and winter seasons for every stixel by predicting at the median date within season (December–February dates were adjusted to a continuous scale).

We derived two measures of thermal sensitivity from partial dependence plots fitted for temperature–occurrence relationships within each stixel: (1) thermal peak, the value of daily temperature at which predicted occurrence is maximized; (2) thermal range, equal to the difference between the value of daily temperature above the thermal peak at which predicted occurrence falls below 80% of the maximum value and the value below the thermal peak at which occurrence falls below 80% of the maximum value. The 80% threshold is in line with many physiological studies measuring thermal optimum and breadth (e.g. [48]).

For both measures, we quantified the regional and seasonal variation in thermal sensitivity within each species by summarizing how thermal peak and range varied across the species range and between seasons. To estimate spatial variation, we regressed mean annual temperature (bio1 from Worldclim [41]) on the thermal peak to calculate the slope across all stixels spanning a geographical–climatic gradient within the given season, summer or winter. Similarly, we regressed mean annual temperature range (bio7) against thermal range to calculate the slope of thermal range spanning a geographical–climatic gradient within the season. A slope closer to 1 suggests that stixel-level thermal peak or range is closely associated with local environmental conditions, while a slope closer to 0 suggests that each is consistent across the species' range. To estimate seasonal variation, we recorded the mean stixel-level difference in thermal peak or range between seasons and computed a Welch's two-sample *t*-test [49] to evaluate whether the difference in thermal peak or range between winter and summer is statistically different. Greater differences suggest greater seasonal variation in thermal response. Thus, we compiled six metrics for each species: spatial (two seasons) and seasonal variation in thermal peak and range.

All plots visualizing metrics were generated using *ggplot2* [50] and *RColorBrewer* [51].

### (f) Spatial predictions

We generated maps depicting spatial variation in thermal response throughout the range of each species across both the winter and summer seasons. First, we created a gridded dataset with 2.8 km^2^ resolution and generated model predictions of occurrence in each cell assuming 12 evenly spaced values of daily temperature ranging between 0° and 36°C, assigning a thermal peak to each cell corresponding to the temperature at which occurrence in the cell was maximized. We then determined thermal range by calculating the number of continuous temperature values at which occurrence was predicted to be 80% of the maximum. We held observation process features constant to remove variation in detectability, resulting in occurrence predictions for a standardized eBird search (an average observer travelling 1 km over 1 h). For each cell, we compiled values of per cent land cover, elevation, and topographic features for use when generating predictions. For each species, we generated these predictions at the hour of the day when the species is most often observed based on our data, and on a day with mean annual 1970–2000 temperatures and total precipitation. Maps were generated using the *purr* package [37] and plotted using *RColorBrewer*.

### (g) Species trait and phylogeny assessment

Our final goal was to determine whether spatial and seasonal variation in thermal sensitivity is associated with avian life-history traits at the species level. We compiled information on preferred habitat (merging forest with woodland and grassland with shrubland categories), body mass (log-transformed) and hand–wing index from AVONET [52]. Further, we calculated species-level landcover diversity index (following [29]) to represent habitat generalism, based on mean partial effects of all landcover features in independent continent-wide SDMs (J. Cohen, W. Jetz 2023, in preparation). Thus, we compiled four traits.

To assess phylogeny as a driver of spatial and seasonal variation in thermal sensitivity, we calculated Blomberg's *K* [53] using an avian phylogeny [54] and comparing it with a null distribution of *K* after randomizing species' responses 1000 times (*picante* package; [55]). Finally, we fitted six multivariate phylogenetic generalized least-squares (PGLS) models to assess the simultaneous influence of traits and phylogeny on each of the six metrics. We propagated error associated with spatial and seasonal variation in thermal sensitivity (s.e. of slope coefficients and confidence intervals associated with *t*-values, respectively) through these models by weighting each estimate in the model by the inverse of the error term. We then fitted ANOVAs to each model to assess the importance of the categorical variable (habitat preference).

Code associated with the study is available via Dryad (https://doi.org/10.5061/dryad.rn8pk0phg) [56] and data is available via www.ebird.org.

## 3. Results

### (a) Do birds vary in thermal sensitivity across their ranges?

**Figure 3.**
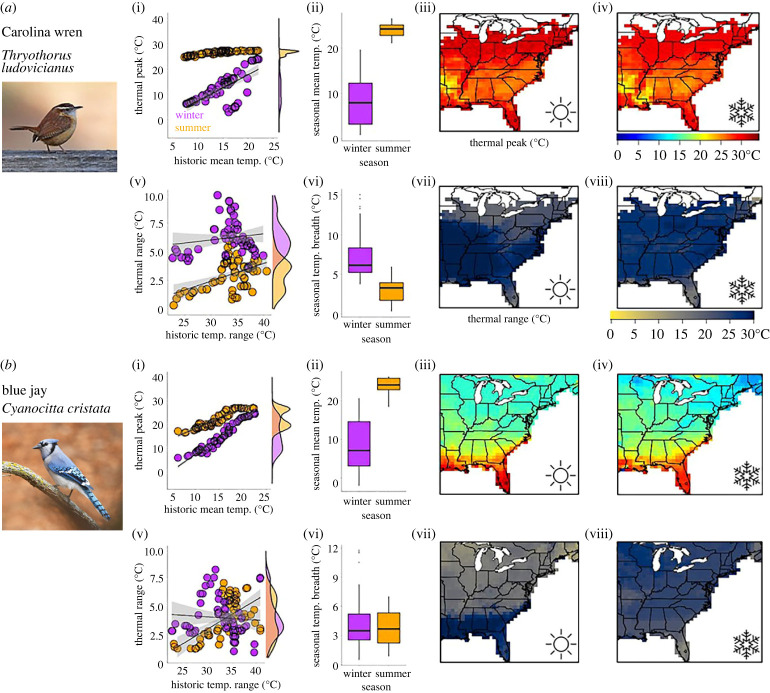
Spatial and seasonal variation in thermal sensitivity differs between species. Scatterplots (i,v) illustrate relationships between annual seasonal mean temperature and thermal peak (the daily temperature at which activity level is greatest in each region based on model predictions; points), or relationships between historic seasonal temperature range and thermal range (the range of temperatures at which activity levels are above 80% of maximum) for each stixel (modelling subunit). Patterns are given across summer (orange points, sun graphic) and winter (purple points, snowflake), with shaded 95% confidence bands. Marginal density plots illustrate seasonal differences in thermal peak and range across stixels, which can be compared against seasonally available mean temperature and temperature range across stixels (ii,vi). Maps (iii,iv,vii,viii) visualize thermal peaks in space for each species across both seasons. (*a*) Carolina wren (*Thryothorus ludovicianus*) has a consistent peak at warm temperatures with moderate spatial variation across the map, with seasonal variation in peak occurring only in cold climates. It has moderate variation in range during both seasons. (*b*) Blue jay (*Cyanocitta cristata*) has high spatial and low seasonal variation in peak, but more seasonal variation in range. Note that patterns in scatterplots may not directly correspond to those on maps because scatterplots summarize thermal sensitivity at the stixel level while maps average multiple (10–20) stixels at the point level.

**Figure 4.**
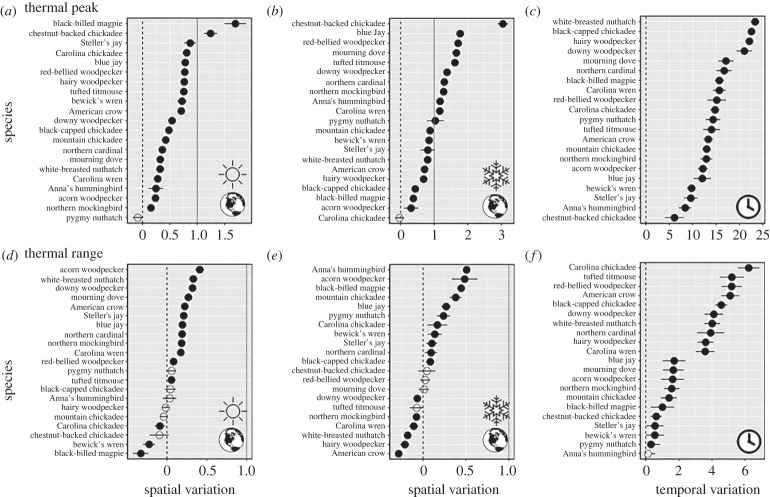
Variation in thermal sensitivity within 21 North American bird species. Estimates of spatial (Earth graphic) and seasonal (clock) variation in thermal peak (*a–c*) and range (*d–f*). Spatial variation is defined as the slope coefficient (±s.e.) describing the regional-scale relationship between a species' thermal peak or range and the regional mean temperature or temperature range and is presented for summer (*a,d*; sun graphic) and winter (*b,e*; snowflake) seasons. Seasonal variation is defined as the mean stixel-level difference in °C (±95% CI) between a species' thermal peak (*c*) or thermal range (*f*) during summer and winter seasons. The black dotted lines correspond to a value of 0, or no relationship between thermal peak/range and local climate, and grey solid lines correspond to 1, or a 1 : 1 relationship, or strong variation within species. Open circles denote species with error overlapping zero. In (*d,e*), negative values not overlapping 0 suggests that the species may have a smaller thermal range in more seasonal regions.

Overall, most species demonstrated spatial variation in thermal peak and range, though the extent of variation differed among species (table 1; figures 3 and 4). During both seasons, species exhibited higher thermal peaks in warmer climates, although this relationship was stronger during winter (summer: mean *β* = 0.59 ± 0.09 s.e.; winter: 1.09 ± 0.14). Birds also exhibited wider thermal ranges in more variable climates (summer: mean *β* = 0.1 ± 0.04; winter: 0.09 ± 0.05). In both summer and winter, all but one bird species exhibited spatial variation in thermal peak (model coefficient ± s.e. > 0) across climate zones. In summer, thermal peaks of two species (10% of species) closely matched their environment (model coefficient >1), but this increased to 11 species (52%) during winter. Evidence for spatial variation in thermal range was mixed, with 52% of species demonstrating variation in winter and 57% in summer (figure 4).

### (b) Do birds vary in thermal sensitivity across summer and winter seasons?

Bird typically exhibited greater peaks (mean sample difference = 14.74°C ±1.01) and narrower ranges (−2.69°C ± 0.43) in summer than in winter. Seasonal variation in thermal range (the difference in thermal range between summer and winter) was observed in all birds but varied in magnitude across species, and seasonal variation in thermal range was observed in all species except for pygmy nuthatch and Anna's hummingbird (figure 4).

### (c) Do species that vary in thermal sensitivity locally also do so seasonally?

**Figure 5.**
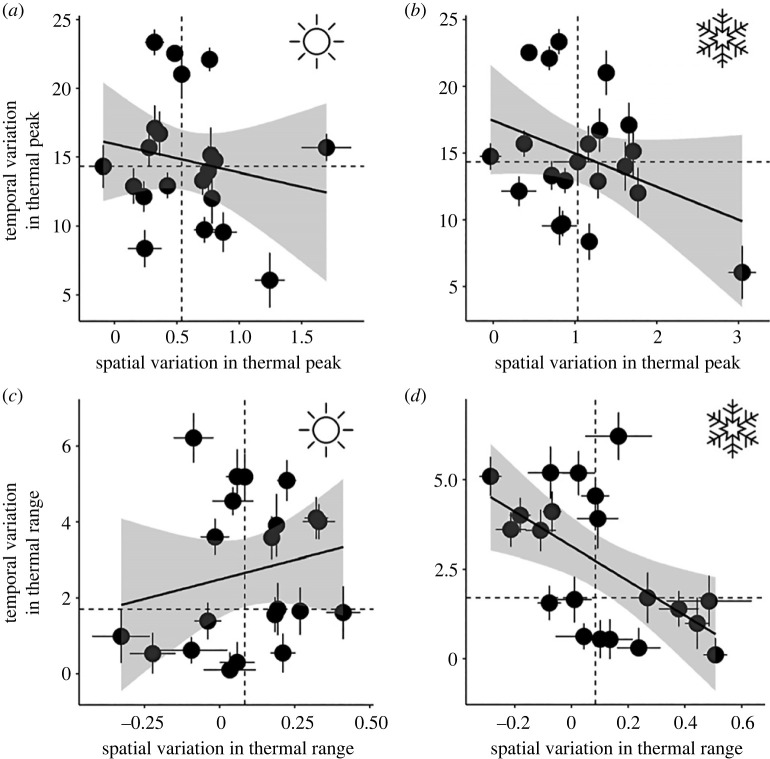
Relationships between spatial and seasonal variation in thermal sensitivity. Spatial variation (*x*-axes), or the slope coefficient (± s.e.) describing the stixel-level relationship between a species' thermal peak or range and the local mean temperature or temperature range, is compared against seasonal variation (*y*-axes), or the mean stixel-level difference in thermal sensitivity across seasons (±95% CI), with points representing species. In (*a,b*), these comparisons are visualized for thermal peaks; in (*c,d*), thermal ranges. (*a,c*; sun graphic) Trends in summer, and (*b,d*; snowflake) trends in winter. All variables were standardized to increase interpretability. Linear trendlines are given with grey shading representing 95% confidence bands. Dashed lines represent medians.

Across species, we observed that birds with greater spatial variation generally had lower seasonal variation, especially in winter (peak, *β* = −2.47 ± 1.50 s.e.; range, *β* = −4.82 ± 1.62; figure 5).

### (d) Is variation in responses to thermal conditions mediated by species' traits or phylogeny?

**Figure 6.**
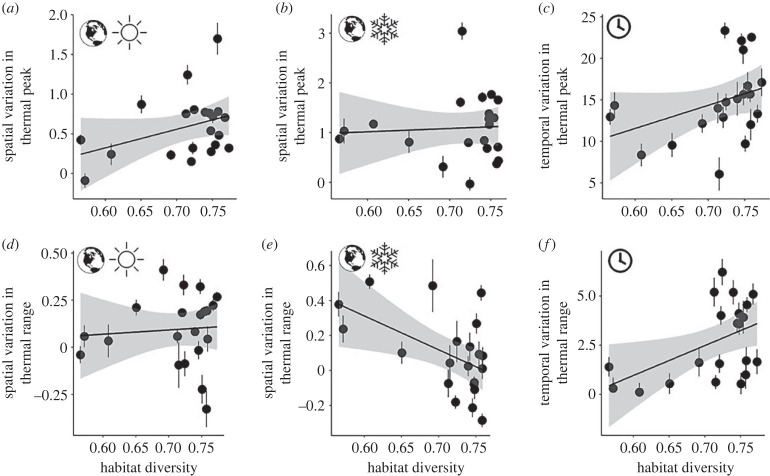
Habitat diversity is associated with the extent of variation in thermal sensitivity within species. At the species level (points), partial residual plots visualize relationships between an index of habitat diversity (*x*-axes) and (*a*) spatial variation (Earth graphic) in summer (sun), or the slope coefficient (±s.e.) describing the stixel-level relationship between a species' thermal peak or range and the local mean temperature or temperature range in thermal peak, (*b*) spatial variation in winter (snowflake), or (*c*) seasonal variation (clock graphic), representing the mean stixel-level difference in thermal sensitivity across seasons (*y*-axes, ±95% CI), based on phylogenetic least-squares models. In (*d–f*), equivalent relationships are presented for thermal range. Linear trendlines are shown with grey shading representing 95% confidence bands.

We found no evidence that phylogeny is associated with spatial or seasonal variation in thermal sensitivity across species (*K* < 0.39, *λ* < 0.32, *p* > 0.1 for all metrics; electronic supplementary material, table S1). Most species traits were not associated with variation either. However, habitat diversity consistently emerged as associated with spatial or seasonal variation in thermal peak and range after controlling for phylogeny. For example, habitat generalists were less likely to exhibit spatial variation in thermal range in winter (PGLS: *β* = −1.69, *p* 0 0.005), while more likely to show seasonal variation in thermal peak (*β* = 40.62, *p* < 0.05) and thermal range (*β* = 18.31, *p* < 0.005; figure 6; electronic supplementary material, tables S2 and S3). We did not detect consistent effects of daily temperature on human observer effort or variation (RMSE = 8.05; Spearman's *ρ* = 0.54; electronic supplementary material, figure S1).

## 4. Discussion

Thermal sensitivity and responses to climate change are typically quantified at the species level [4], but recent evidence suggests significant physiological and morphological variation within species, likely as a result of local adaption and phenotypic plasticity [6–8,12]. Researchers require a better understanding of variation in thermal sensitivity within species to assess when and where populations are more likely to be sensitive to weather-related effects [4,7,9]. However, thermal sensitivity is difficult to measure across numerous populations and multiple seasons for many species. Here, we use an ensemble modelling approach with multiple dynamic SDMs that allows spatial and seasonal variation in temperature responses to identify patterns of spatial and seasonal variation in thermal sensitivity across common North American resident birds. We found that birds may exhibit high or low levels of intraspecific variation in responses to temperature across space and time, suggesting that local adaptation and phenotypic plasticity are more important for some species than others. Importantly, typical SDMs would have estimated a constant relationship between temperature and occurrence for each species, missing important heterogeneity in this relationship over both their geographical distributions and the annual cycle.

Our findings support physiological work [16,24] suggesting that populations of a species vary in their sensitivity to thermal conditions based on geography. For both thermal peak and range, most species occupied an intermediate space between a lack of intraspecific variation, or high variation among locations that perfectly matches the local environment. Thermal peak was more likely than thermal range to match local environmental conditions, with 91% of species (19 of 21) demonstrating a relationship between thermal peak and local climate, and only 52% (11 of 21) demonstrating such a relationship for thermal range. In fact, for 10 of 21 species (48%) thermal peaks more closely matched the local environment than conspecifics in different regions (i.e. local adaptation)—however, only 2 of 21 species (9.5%) demonstrated thermal sensitivity that perfectly matches the local environment. It has long been known that thermal range is highly important in terms of constraining organismal distributions, likely more so than thermal peak [57,58], and our results may suggest that thermal range is a more hardwired physiological constraint than thermal peak across populations of many bird species. Across species, we found that spatial variation in thermal sensitivity was infrequently associated with phylogeny or species traits, although the limited sample of 21 species constrained our ability to draw broad inferences. We also found limited evidence that spatial variation in thermal range was greater in habitat generalists than in specialists. This link was predicted because habitat and thermal generalism are often observed in the same species [28], and thermal generalists may be more likely to adapt to the local environment.

Surprisingly, all species reflected different thermal peaks, and 86% (18 of 21) demonstrated different thermal ranges across seasons, despite substantial overlap in conditions across seasons in most species' geographical distributions. However, this pattern may not be representative of all bird species; the species in our selection are mostly residential and thus more likely than other bird species to be seasonally flexible in thermal sensitivity. Interestingly, habitat generalism was more closely associated with seasonal variation in both thermal peak and range. Therefore, habitat generalists may be selecting a strategy in which they eschew local adaptation in favour of seasonal flexibility across the annual cycle resulting from phenotypic plasticity. Finally, during winter, species with greater spatial variation in thermal sensitivity had reduced seasonal variation, suggesting a trade-off; for example, a species with populations that are highly locally adapted to climate may not need to rely as heavily on phenotypic plasticity.

Local adaptation over space and phenotypic plasticity over seasons may be more difficult to study (using our approach) in species that seasonally move long distances, occupy smaller geographical distributions, or are reported less frequently. Thus, we avoided such species in our study. Within species that seasonally migrate long distances, seasonal variation in thermal sensitivity is difficult to measure because, without knowing which sets of locations have the same individuals (e.g. information on migratory connectivity; [59]), direct comparisons between populations over time are difficult. However, recent improvements in animal tracking, even for smaller birds, will allow direct comparisons of thermal sensitivity at the population or individual level even for migratory species [60]. Genetic evidence suggests that the northernmost breeding populations of yellow warblers (*Setophaga petechia*) overwinter at the northern edge of the nonbreeding range [61], although this rule may not be reliable for species that compress their ranges during winter, as do many neotropical migrants [62]. Species that occupy small geographical areas may exhibit little spatial variation in thermal sensitivity, as climate generally varies across large spatial scales. Although local adaptation to different climates is possible along elevational gradients, differences in data abundance between lowlands and uplands may inhibit direct comparisons between adjacent populations inhabiting each zone. Finally, assessing variation in thermal sensitivity may be more difficult for species with limited data coverage in space and time, including birds outside North America or most other animal taxa, although citizen science observations are increasing exponentially every year [63]. Despite these limitations, our results provide a framework to predict how widespread, residential species with continuous data coverage may vary in population and seasonal thermal sensitivity at fine scales.

Although bird species varied in their extent of spatial and seasonal variation in thermal sensitivity, and thus likely their local adaptation and phenotypic plasticity, it remains unclear how this translates to climate change vulnerability. Plausible explanations exist for positive or negative relationships between intraspecific variation in thermal sensitivity and climate change vulnerability. For example, a species exhibiting local adaptation to climate may be more vulnerable to climate change if populations are adapted to distinct thermal conditions and some regions warm faster than others (e.g. northern latitudes warming faster than southern latitudes). Given local adaptation, a continent-wide heat wave may pose a greater risk of disturbance to a northern population of a given species if it has less heat tolerance than a southern population. Alternatively, populations of a species exhibiting no variation over space may be more vulnerable if southern populations already living on the edge of their thermal tolerance experience an extreme weather event, such as a heat wave. Such species cannot benefit from portfolio effects, in which sufficient phenotypic variability allows some populations to persist in the face of disturbance [64]. Future work should explore how variation in thermal sensitivity along a climatic gradient is related to population-level consequences to aid finer-scale conservation approaches.

## 5. Conclusion

Researchers typically predict and measure static responses to climate change at the species level [4], while conservationists and managers typically develop climate change vulnerability assessments and adaptation plans for species, ignoring population-level variability. However, with the modern availability of high-resolution, high-volume, continuous observational and environmental datasets, variation in species' responses to environmental variables, such as temperature, can now be modelled over large spatial extents and across the annual cycle to detect variation in responses to climate change at higher resolutions [65,66]. Our results suggest that many species-level assessments of thermal sensitivity may be missing significant local adaptation among populations and phenotypic plasticity within individuals, leading to misleading vulnerability assessments. Researchers must consider variation in thermal sensitivity across populations and seasons to improve understanding of climate change adaptation [4].

## Data Availability

The code supporting the results is available from Dryad (doi:10.5061/dryad.rn8pk0phg) [56] and the data are accessible via http://www.ebird.org. Supplementary material is available online [67].
